# Literary Notices

**Published:** 1876-09

**Authors:** 


### LITERARY NOTICES.

*Scribner's Illustrated Monthly* for October, is one of the most charming numbers of this superb magazine. The illustrations are full of interest—they are always so in *Scribner's*—and the literary matter is pure and elevated. The high moral tone of the editorial department is in keeping with the past history of the *Monthly*, and always shows the care and literary *spice* of Dr. Holland. We know of no greater treat—no better literary feast —for the home circle than *Scribner.* It will delight the young and the old, and be treasured for years, if bound and placed in the library.

